# Understanding Clinicians’ Adoption of Mobile Health Tools: A Qualitative Review of the Most Used Frameworks

**DOI:** 10.2196/18072

**Published:** 2020-07-06

**Authors:** Christine Jacob, Antonio Sanchez-Vazquez, Chris Ivory

**Affiliations:** 1 Anglia Ruskin University Cambridge United Kingdom; 2 University of Applied Sciences Northwestern Switzerland Brugg Switzerland; 3 Innovation and Management Practice Research Centre Anglia Ruskin University Cambridge United Kingdom

**Keywords:** telemedicine, smartphone, electronic health record, workflow, workload, workplace, public health practice, technology, perception, health education, mHealth, mobile health, telehealth, eHealth

## Abstract

**Background:**

Although there is a push toward encouraging mobile health (mHealth) adoption to harness its potential, there are many challenges that sometimes go beyond the technology to involve other elements such as social, cultural, and organizational factors.

**Objective:**

This review aimed to explore which frameworks are used the most, to understand clinicians’ adoption of mHealth as well as to identify potential shortcomings in these frameworks. Highlighting these gaps and the main factors that were not specifically covered in the most frequently used frameworks will assist future researchers to include all relevant key factors.

**Methods:**

This review was an in-depth subanalysis of a larger systematic review that included research papers published between 2008 and 2018 and focused on the social, organizational, and technical factors impacting clinicians’ adoption of mHealth. The initial systematic review included 171 studies, of which 50 studies used a theoretical framework. These 50 studies are the subject of this qualitative review, reflecting further on the frameworks used and how these can help future researchers design studies that investigate the topic of mHealth adoption more robustly.

**Results:**

The most commonly used frameworks were different forms of extensions of the Technology Acceptance Model (TAM; 17/50, 34%), the diffusion of innovation theory (DOI; 8/50, 16%), and different forms of extensions of the unified theory of acceptance and use of technology (6/50, 12%). Some studies used a combination of the TAM and DOI frameworks (3/50, 6%), whereas others used the consolidated framework for implementation research (3/50, 6%) and sociotechnical systems (STS) theory (2/50, 4%). The factors cited by more than 20% of the studies were usefulness, output quality, ease of use, technical support, data privacy, self-efficacy, attitude, organizational inner setting, training, leadership engagement, workload, and workflow fit. Most factors could be linked to one framework or another, but there was no single framework that could adequately cover all relevant and specific factors without some expansion.

**Conclusions:**

Health care technologies are generally more complex than tools that address individual user needs as they usually support patients with comorbidities who are typically treated by multidisciplinary teams who might even work in different health care organizations. This special nature of how the health care sector operates and its highly regulated nature, the usual budget deficits, and the interdependence between health care organizations necessitate some crucial expansions to existing theoretical frameworks usually used when studying adoption. We propose a shift toward theoretical frameworks that take into account implementation challenges that factor in the complexity of the sociotechnical structure of health care organizations and the interplay between the technical, social, and organizational aspects. Our consolidated framework offers recommendations on which factors to include when investigating clinicians’ adoption of mHealth, taking into account all three aspects.

## Introduction

### Background

Health care sectors worldwide are witnessing the rapid emergence of new tools using mobile technology to support medical practice; new apps are created and launched daily. The World Health Organization global observatory of eHealth defines mobile health (mHealth) as “medical and public health practice supported by mobile devices, such as mobile phones, patient monitoring devices, Personal Digital Assistants (PDAs), and other wireless devices.” Telemedicine itself is a growing and well-established subcategory of mHealth and is defined as “the communication or consultation between health professionals about patients using the voice, text, data, imaging, or video functions of a mobile device. But it can be applied to other situations; the management of chronic diseases of patients living at home is one example” [1].

Previous research has shown that such tools have promising potential. They can save time and improve efficiency [2], increase patient access and decrease workload [3], empower patients and address their information needs [4], enhance patient-clinician communication by prompting some underreported and sensitive discussions [5,6], and make patients feel more taken care of [5,6]. The patient data generated through some of these tools can also help care teams to prioritize and tailor treatment plans accordingly [5]. Research has also shown that the potential benefits encourage even older populations to adopt such tools [7] despite the widespread notion of a *digital divide* and boost patients’ quality of life through tailored and personalized treatment plans [4,5].

However, it remains that the dynamic and liberal way in which the mHealth market is organized makes it difficult to assess the quality of the tools on offer, making adoption decisions more difficult, especially from the perspective of a clinician [8]. In addition, the amount of health data generated by such apps creates a need for more comprehensive privacy regulations, in particular, around the use of personal data that are clinically and scientifically meaningful [9,10]. Furthermore, high workload, lack of resources, and necessary workflow adjustments were frequently raised as barriers to adoption [11-16].

### Objective

Although there is a drive toward encouraging mHealth adoption to harness its potential benefits, the reality is that there are many challenges that go beyond the technology itself but stem instead from the complexity of the health care ecosystem itself. Such challenges have slowed down the acceptance and adoption of mHealth in specific health care settings [17,18], with some failing to progress beyond the pilot stage [19] or failing to become a part of the regular care landscape [20,21]. To fully account for the adoption and nonadoption of technology, it is thus essential to shift our focus beyond the technology itself to tackle clinicians’ differing concerns, improving clinical workflow, and workload issues [18,22,23]. Therefore, this review aimed to identify the frameworks most used to successfully understand clinicians’ adoption of mHealth to enable future researchers to design studies that take all the important factors into account, leading to more successful adoption and implementation.

We were guided in our review by the field of social studies of technology that views individuals and technological artifacts as entangled and interacting elements in any organizational or social setting [24-27], bearing in mind that such interactions may trigger or enable new forms of organizing work, new roles, or new hierarchies [28,29]. This ontological approach enabled us to widen our scope and identify potential shortcomings in the most frequently used frameworks. We found that the most frequently applied models tend to focus on the technical or individual perspectives of technological adoption.

## Methods

### Search Strategy and Study Selection

This review was an in-depth subanalysis of a larger systematic review focusing on the social, organizational, and technical factors impacting clinicians’ adoption of mHealth [30]. The Preferred Reporting Items for Systematic Reviews and Meta-Analyses guidelines [31] and the Cochrane handbook [32] were followed to ensure a systematic and rigorous approach. A structured search was carried out using Medical Literature Analysis and Retrieval System Online, or MEDLARS Online (MEDLINE), PubMed, the Cochrane Library, and Serial Analysis of Gene Expression (SAGE) databases for studies published in the English language between January 2008 and July 2018, yielding 4993 results. Of these 4993 studies, 171 met the inclusion criteria, of which only 50 studies used a theoretical framework. These latter 50 studies were the subject of this qualitative review. The review presented here reflects more deeply on the most common frameworks used and on how researchers can be supported to design more robust and reliable studies that properly investigate and reflect the inherent complexity of the issue of mHealth adoption.

### Data Collection and Synthesis

The diversity of measures and results that were identified in the studied sample was not homogenous enough to enable a quantitative synthesis of the data. Therefore, a narrative synthesis was used and structured around the organizational, social, and technological factors impacting the clinician's adoption of mHealth solutions. NVivo (QSR International), a computer-assisted qualitative data analysis software, was used to assist in this task.

Data coding began with an initial data extraction grid that included themes based on the most used technology acceptance frameworks in the studied sample; more themes were added as they emerged during the review process. A thematic analysis as per Braun and Clarke [33] was used to identify and extract themes that addressed the review’s research question; the phases of the thematic analysis are explained in detail in Multimedia Appendix 1. The first author (CJ) conducted the initial analysis and coding; she is a digital strategist with more than 18 years of experience and has contributed to the creation and realization of several digital solutions in health care. Then, the second author (ASV) reviewed the coding; any cases of disagreement about coding were discussed in conjunction with the last author (CI) and mutually agreed.

The research themes were guided by the most used theoretical frameworks in the studied sample, in addition to Leonardi’s *Methodological Guidelines for the Study of Materiality and Affordances* [27]; hence, they were split into three key groups: material and technological factors, social and personal factors, and organizational and policy factors. This process lasted from August to December 2019.

## Results

A summary of each of the included studies, the theoretical framework(s) they used, sample size, sample composition, data collection method, and key findings are detailed in Multimedia Appendix 2.

### Characteristics of the Included Studies

The sample characteristics of the included papers are detailed in Multimedia Appendix 3. In terms of data collection methods, 56% (28/50) of the studies used quantitative methods, 26% (13/50) of the studies used qualitative methods, and 18% (9/50) of the studies used a mixed approach. Altogether, 42% (21/50) of the studies focused on clinicians (these were studies that included multiple clinician groups, with a clinician defined as “a person qualified in the clinical practice of medicine, psychiatry, or psychology as distinguished from one specializing in laboratory or research techniques or in theory” [34]), 30% (15/50) of the studies focused on physicians, 10% (5/50) of the studies focused on nurses, 4% (2/50) of the studies focused on medical students, and 14% (7/50) of the studies focused included multiple clinician groups and other populations such as patients or caregivers. Sample sizes varied, that is, 18% (9/50) of the studies had a sample size of 1 to 20 participants, 18% (9/50) of the studies had a sample size of 21 to 50 participants, 20% (10/50) of the studies had a sample size of 51 to 100 participants, 28% (14/50) of the studies had a sample size of 101 to 200 participants, and 16% (8/50) of the studies had a sample size of more than 200 participants.

Geographically, 24% (12/50) of the studies were based in the United States [35-46], 10% (5/50) of the studies were based in the United Kingdom [47-51], and the rest were spread across many countries: Australia [52,53], Australia and the United Kingdom [15], Austria [54], Canada [55-57], Germany [58-60], Iran [61], Iraq [62], Japan [63], the Netherlands [64], the Netherlands, Spain, and the United Kingdom [65], Nigeria [66], Norway [67,68], South Korea [69,70], Spain [71-74], Spain Colombia and Bologna [75], Sri Lanka [76], Sweden [77,78], Switzerland [79], Taiwan [80,81], and Turkey [82].

The studies also cover diverse disease areas and medical specialties, including ambulatory care [58], cognitive behavioral therapy [40], cardiovascular disease [56,83], dermatology [73], diabetes [50,63,65], general practice [35,52,59], intensive care [36], neurology [48], pediatric [45], primary and acute care [46,49,55,71,74], psychiatry and mental health [41,53,68], residential aged care [57], reproductive health [37], and substance use recovery [39,44].

### Frameworks Mostly Used in Studying Clinicians’ Adoption of Mobile Health

The most commonly used frameworks were different forms of extensions of the Technology Acceptance Model (TAM), the diffusion of innovation theory (DOI), and different forms of extensions of the unified theory of acceptance and use of technology (UTAUT). An overview of the most used frameworks is shown in Table 1 and visualized in Multimedia Appendix 4.

**Table 1:** Overview of the most used frameworks.

Framework(s)	Prevalence in the studied sample (N=50), n (%)
TAM^a^ extensions	17 (34)
Others	11 (22)
DOI^b^	8 (16)
UTAUT^c^ extensions	6 (12)
DOI and TAM	3 (6)
CFIR^d^	3 (6)
Sociotechnical theory	2 (4)

^a^TAM: Technology Acceptance Model.

^b^DOI: diffusion of innovation theory.

^c^UTAT: unified theory of acceptance and use of technology.

^d^CFIR: consolidated framework for implementation research.

TAM extensions were used in 34% (17/50) of the studies and varied from additions stemming from the literature and the research-specific context [35,36,38,57,60,61,68,70,71,73,74] to extensions using other frameworks such as Chau and Hu’s model of telemedicine acceptance and theory of interpersonal behavior (TIB) [83], the organizational readiness for change model [45], theory of reasoned action (TRA), and theory of planned behavior [74], and in combination with the UTAUT [58,69].

DOI was used in 16% (8/50) of the studies [37,40,50,53,56,62,76,78], and UTAUT extensions were used in 12% (6/50) of the studies; some of these UTAUT extensions were based on the research context and previous research [64,66,84], and other included extensions from other frameworks such as the De Lone and McLean information success model [54], use of technology [65], and a combination of TAM, TPB, and DOI [82]. Furthermore, certain studies used a combination of the TAM and DOI frameworks (3/50, 6%) [42,43,59]; others used the consolidated framework for implementation research (CFIR; 3/50, 6%) [39,41,67] and the STS (2/50, 4%) [49,55].

Other frameworks were used in 22% (11/50) of the studies, including the affordability, practicability, effectiveness, acceptability, side effects/safety and equity (APEASE) framework [52]; an extended De Lone and McLean information system success model [63]; Giddens structuration theory [51]; normalization process theory (NPT) [15]; organizational theory of implementation effectiveness [46]; reach, effectiveness, adoption, implementation, and maintenance framework [44]; technological frames (TF) [77]; the design science research methodology (DSRM) [85]; theory of change [48]; and TPB [80].

### Framework-Based Versus Additional Factors

We will not present an analysis of each factor in detail, as this was extensively covered in the initial systematic review [30]; we will instead focus on the framework-based versus additional factors to better identify possible shortcomings in the most used frameworks and provide recommendations based on the specificities of the health care setting, as reported in the studied sample.

**Figure 1:**
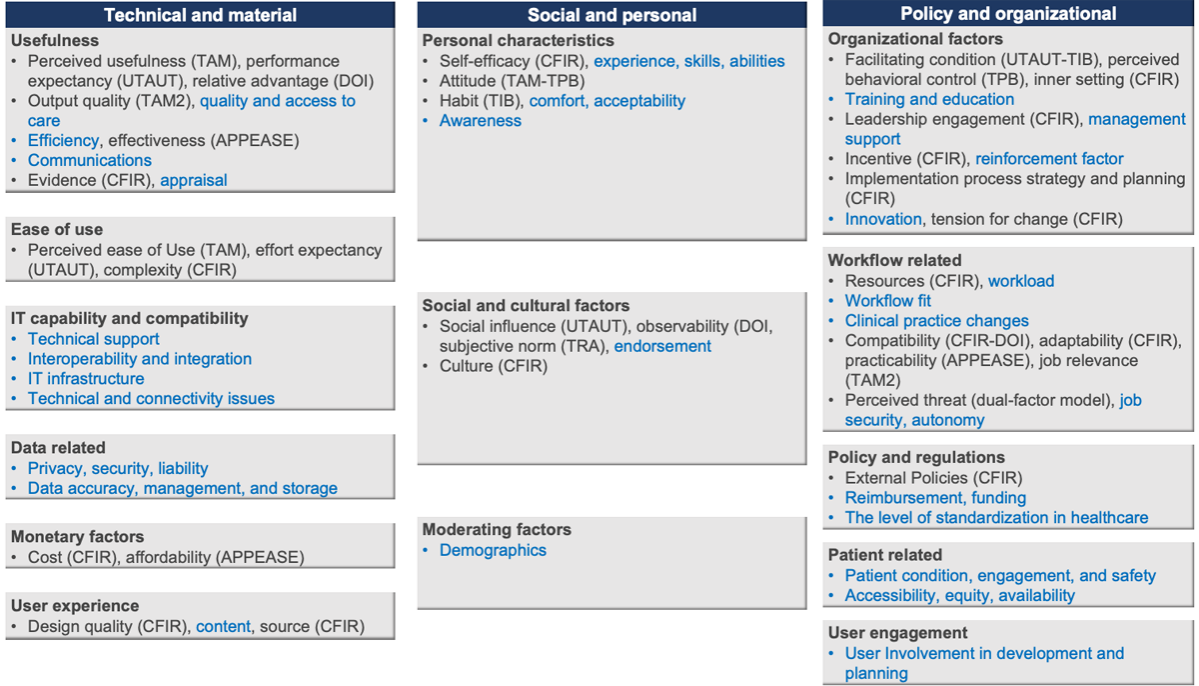
Gap analysis of emerging framework-based versus additional factors. APEASE: affordability, practicability, effectiveness, acceptability, safety/side effects, and equity; CFIR: consolidated framework for implementation research; DOI: diffusion of innovation theory; IT: information technology; TAM: Technology Acceptance Model; TAM2: an expanded version of the original TAM; TPB: theory of planned behavior; TRA: theory of reasoned action; UTAUT: unified theory of acceptance and use of technology.

Factors cited in more than 20% of the included studies were usefulness, output quality, ease of use, technical support, data privacy, self-efficacy, attitude, organizational inner setting, training, leadership engagement, workload, and workflow fit. Most factors could be linked to one framework or another, but there were no frameworks that could cover all factors in our findings unless modified or used in combination with others. An overview of the emerging framework-based and additional factors is presented in Figure 1. Factors defined as specific constructs in one of the used frameworks include the name of the framework following the factor name between brackets, whereas the factors that emerged directly from the data but were not identified as a specific construct in any of the used frameworks are not followed by brackets and are marked in blue font for clarity. These additional factors stemmed mostly from qualitative studies or were predefined by the researchers’ review of previous literature findings rather than established frameworks.

Some factors with more or less the same meaning were named differently in different frameworks. For example, when we talk about the tool’s usefulness, it can be referred to as perceived usefulness if the study uses TAM, as performance expectancy if the UTAUT is used, or as relative advantage if DOI or CFIR is used. Similarly, when we talk about the tool’s ease of use, it can be referred to as perceived ease of use if the study uses TAM, as effort expectancy if the UTAUT is used, or as complexity if DOI or CFIR is used. We grouped these similar factors together in our analysis to avoid an overly congested schema of factors. Multimedia Appendix 5 summarizes the definitions of the most used framework-based factors grouped by similarity to emphasize the meaning and overlaps between the different theories.

### Technical and Material Factors

**Figure 2:**
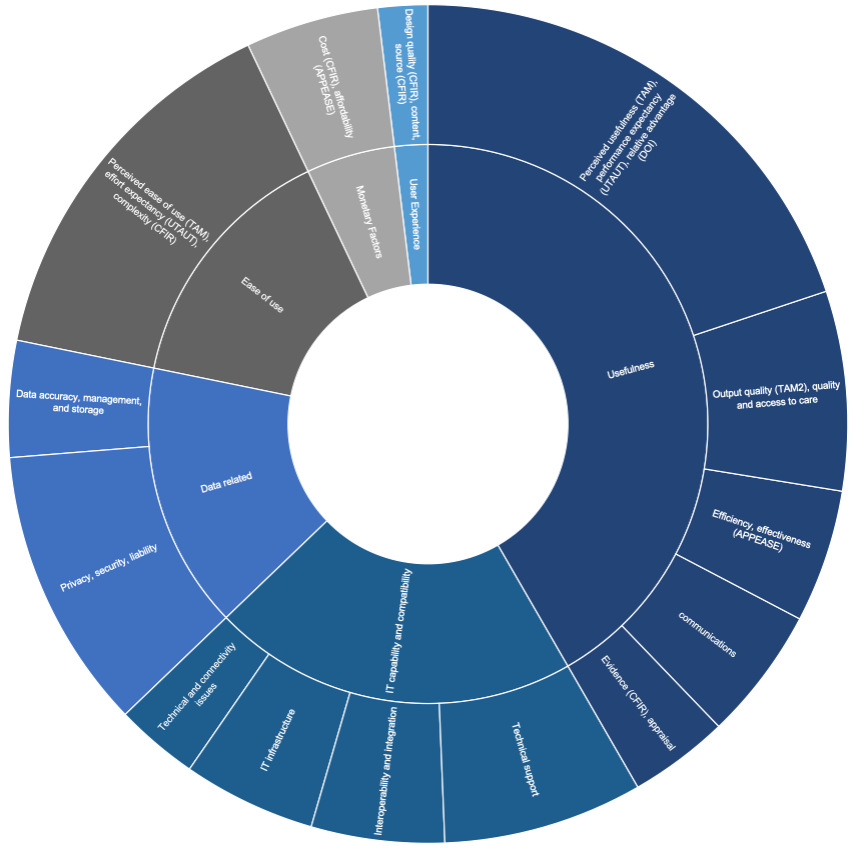
The most cited technical and material factors. APEASE: affordability, practicability, effectiveness, acceptability, safety/side effects, and equity; CFIR: consolidated framework for implementation research; DOI: diffusion of innovation theory; IT, information technology; TAM: Technology Acceptance Model; TAM2: an expanded version of the original TAM; TPB: theory of planned behavior; TRA: theory of reasoned action; UTAUT: unified theory of acceptance and use of technology.

When we look into the most reported technical and material factors, as visualized in Figure 2, we can see that the clusters of ease of use and usefulness were the most reported in the included papers; however, other important acceptance elements such as health data–related factors and system interoperability may be strong influencing aspects, but these are not specifically considered in most of the original models.

The cluster of usefulness factors tops the list of technical and material factors. Most studies (n=31) reported usefulness, in general, using theory-based constructs such as perceived usefulness (TAM), performance expectancy (UTAUT), or relative advantage (DOI). Many of these studies, especially those that mainly relied on TAM, did not necessarily dig into the hidden elements that might influence the views of users on usefulness; yet, some still attempted to dig deeper to get at the underlying factors that influence perceived usefulness. For example, Gagnon et al [83] and Orruno et al [73] assumed that compatibility with one’s work would impact the perception of a tool’s usefulness, whereas Liu and Cheng [81] anticipated that a user’s perception that a tool is a threat to their job would also impact their perceived usefulness, and Ray et al [45] proposed that there is a whole cluster of contextual elements such as organizational and individual factors that may impact perceived usefulness. Most of the studies found that usefulness is one of the most if not the most influential factor when it comes to adoption decisions [36,66,69,71,83], whereas other studies reported that a lack of perceived advantage of the system would be considered a barrier to adoption [50].

Output quality (an additional factor in the expanded version of the original TAM [TAM2]) or quality and access to care were more specific expressions of usefulness, as reported in 12 studies. This is a health care specific factor, as mHealth was seen as the most useful and adding value when it enhances patient care [42,46,67] and facilitates access to care for people who would not otherwise access it easily [45,53]. Moreover, eight studies named increased efficiency or effectiveness (APEASE) as a facilitating factor, as it allows optimal use of staff time [47,50], but some also reported mixed views on whether such tools can really improve efficiency and the necessity of streamlining processes to enable such gains [35,52]. Similarly, eight studies specified communication; however, some saw it as a facilitator [42,47,50], and others reported the risk of miscommunication as a barrier [51,53,76], whereas six studies specified evidence (CFIR) or appraisal, showing that growing the evidence base and the *positive stories* about such tools can help increase adoption [39,48,53].

The ease of use cluster (n=23) was named differently in different theories: perceived ease of use in TAM, effort expectancy in UTAUT, and complexity in CFIR. Similar to the general factor usefulness, *ease of use* lacks the specification of what makes a tool easy to use, making it difficult to understand the hidden factors that can influence a user’s perception of ease of use. This pushed several researchers to operationalize this generic factor by breaking it down into more explicit elements; for example, Gagnon et al [83] and Asua et al [71] assumed that factors such as users’ habits (ie, behaviors that became automatized because of a person’s habit of doing it repetitively) could influence their perception of a tool’s ease of use; whereas Liu and Cheng [81] anticipated that mobility (location flexibility) could impact perceived ease of use. Furthermore, Ray et al [45] proposed that it is influenced by a whole cluster of contextual factors as well as elements such as resource availability, whereas Rho et al [70] proposed that it is impacted by self-efficacy and accessibility of medical records, and Sezgin et al [82] suggested that elements such as demonstrability of results, personal innovativeness, mobile anxiety (fear of telephones), mobile self-efficacy, and habit can have an impact on users’ effort expectancy.

The cluster information technology (IT) capability and compatibility includes factors such as technical support (n=12), interoperability and integration (n=8), IT infrastructure (8), and technical and connectivity issues (n=5). These factors can clearly influence adoption; however, they were not specifically spelled out in the frameworks used. The availability of good technical support and collaboration with the IT department may enable better adoption [44,46,47,71,73,79,82,83], whereas the lack of technical support was reported as a barrier [51,68]. Similarly, if the tool is not integrated into or interoperable with the hospital or clinical systems, it becomes harder to adopt it as it usually creates an increased workload (eg, inputting data), and therefore, the potential for information to be lost [44,47,53,78]. Several studies also reported that an insufficient IT infrastructure could be a barrier [42,53,62,73,76,83] and some specified difficulties such as technical and connectivity issues that seem to be a common problem in some hospitals [50,62,67,68,79].

Similarly, the data-related factors cluster such as data privacy and security (n=17), and elements such as data accuracy, management, or storage (n=7) can clearly impact adoption in the health care setting but were not specifically defined as distinct constructs in the frameworks used. Such factors are quite specific to the health care ecosystem and are crucial for successful implementation. This is mainly driven by the highly regulated nature of the health care sector and the sensitivity of health data that pushes clinicians to be extra cautious when dealing with any system that captures or stores patient data, highlighting the importance of data privacy and security concerns [37,47,53,56,58,62,75], patient confidentiality [48,55,65,68], and the related medicolegal liability [35,42,44].

Monetary factors such as costs (CFIR) and affordability (APEASE) were reported in eight studies; cost, financial constraints, and uncertainty of future funding were usually perceived as barriers to sustained implementation [39,53,64]. User experience factors such as design quality (CFIR), content, and source (CFIR) were mentioned in three studies; they show that aspects such as the trustworthiness of the tool’s content and where it is coming from may influence adoption [54,55,67].

### Social and Personal Factors

The personal characteristics of adopters were reported in more studies compared with social and cultural factors. Aspects such as self-efficacy (CFIR) were reported in 16 studies, highlighting the importance of the clinicians’ previous experiences, technical skills, knowledge, and abilities in general [36,39,42,53,58,59,64,70,85]. These factors are often linked to the users’ perceived ease of use [61,82]. Similarly, attitude (TAM-TPB) was reported in 16 studies, demonstrating the significance of how the positive or negative feelings users have about using such tools may influence their decision to adopt [36,41,42,53,59,62,69,75,80]. Nevertheless, it was also reported that a positive attitude is not enough to ensure adoption [73]. The importance of factors such as habit (TIB), comfort, and acceptability was reported in six studies and can be influenced by other elements such as trust and mobile use in personal life [15,52,55]. It was also reported that anxiety in using mHealth tools might influence adoption negatively [82].

Social factors (n=10) such as the influence of others on a user’s decision to adopt or the extent to which users believe that people who are important to them will approve their adoption of a particular behavior were taken into account in some of the frameworks used under different names: social influence (UTAUT), observability (DOI), subjective norm (TRA), and endorsement. This shows that factors such as linking the app to a reputable organization, coworkers, opinion leaders, image within the organization, and observing other clinicians using such tools may impact adoption [43,57,63-65,74,78,79]. Conversely, some studies found the influence of such social factors to be insignificant [66]. At the same time, culture (CFIR) came up in four studies, explaining how a conservative or risk-averse culture could be a barrier to adoption [48,53,62,67].

**Figure 3:**
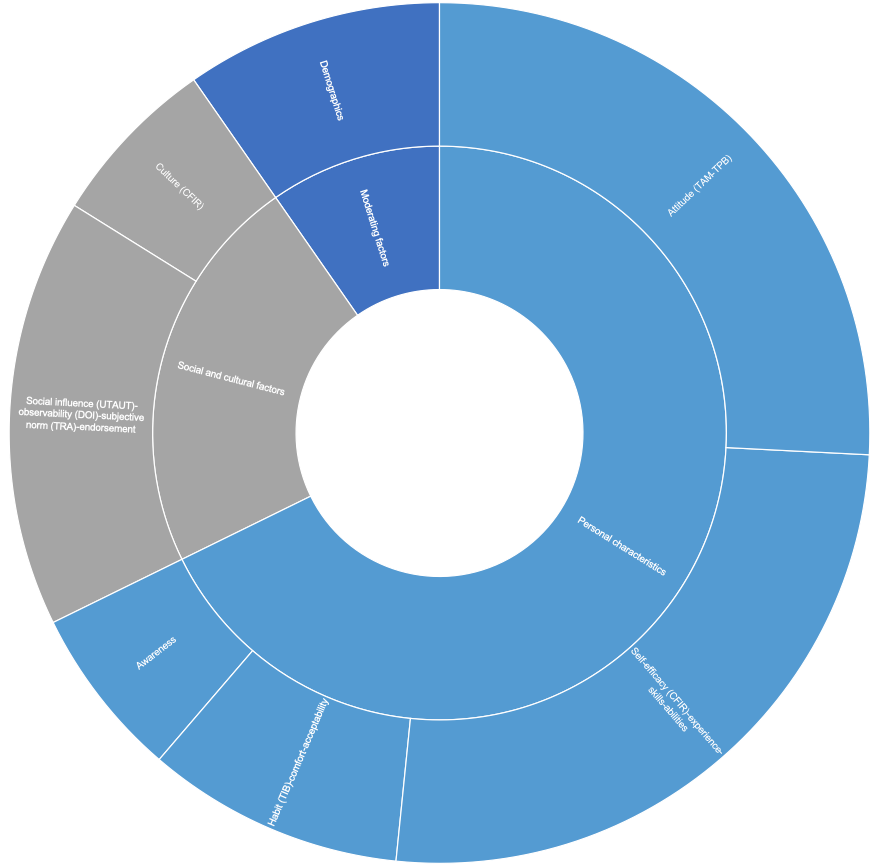
The most cited social and personal factors. CFIR: consolidated framework for implementation Research; DOI: diffusion of innovation theory; TAM: Technology Acceptance Model; TIB: theory of interpersonal behavior; TPB: theory of planned behavior; TRA: theory of reasoned action; UTAUT: unified theory of acceptance and use of technology.

Although demographics are not an initial construct in any of the frameworks used, there were mixed results in the studies that used them as moderating factors (n=6). There were studies that found that elements such as age or gender may have an impact on the decision to adopt [37,59,60], whereas some studies did not find personal demographics to be a significant factor [42,43,64]. An overview of the social and personal factors is shown in Figure 3.

### Organizational and Policy Factors

**Figure 4:**
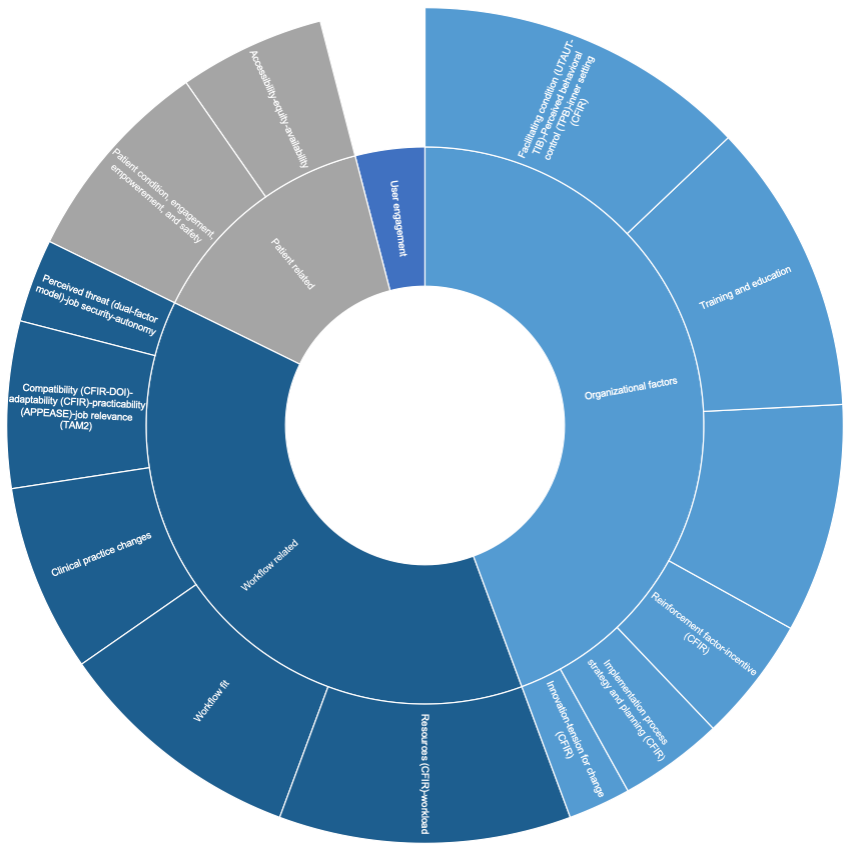
The most cited organizational and policy factors. APEASE: affordability, practicability, effectiveness, acceptability, safety/side effects, and equity; CFIR: consolidated framework for implementation research; DOI: diffusion of innovation theory; TAM2: an expanded version of the original Technology Acceptance Model; TIB: theory of interpersonal behavior; TPB: theory of planned behavior; TRA: theory of reasoned action; UTAUT: unified theory of acceptance and use of technology.

As shown in Figure 4, the organizational and policy factors were divided into 5 key clusters: organizational, workflow-related, policy and regulations, patient-related, and user engagement factors.

Organizational factors (n=16) were taken into account in some of the frameworks used under different names: facilitating conditions (UTAUT-TIB), which is the users’ perception that organizational support and technical facilitating conditions are in place to aid technology use; perceived behavioral control (TPB) designating the perceptions of the availability of skills, opportunities, and resources required for using technology; and inner setting (CFIR) defining the qualities of the organization in which the intervention is implemented. Many studies reported that such organizational facilitators are among the most important factors influencing adoption [41-43,53,65,66,71,73,79,80,83] and explained how the lack of such conditions can hinder adoption [48,67]. More specific factors such as training and education were also explicitly cited in 14 studies [15,39,41,64,68,71,73,82,83], showing their importance, but were not spelled out as a specific construct in most of the frameworks used.

Leadership engagement (CFIR) and management support were other important factors identified in 11 studies. The findings show that top management approval, engagement, and support [15,39,41,47,53,62,67], as well as professional organizations and societies [85] are vital for implementation success and adoption. Reinforcement factors and incentives (CFIR) were reported in six studies as elements that may positively impact adoption [45,48,66,70]. Implementation process, strategy, and planning (CFIR) was reported in five studies, including ideas such as a step-wise approach or piloting the tools before a complete rollout [53,56,58,67]. Factors such as innovation and tension for change (CFIR), which indicate the degree to which stakeholders perceive the current situation as intolerable or needing change, were mentioned in three studies [49,67,75].

The workflow-related cluster was separated from the other organizational factors because of the many factors relating to workflow, which necessitated distinguishing them and highlighting their specificity. Although resource, in general, is a construct in the CFIR, workload was more specifically mentioned in many of the included studies (n=14), showing that an elevated workload or the perception that such tools would increase workload may hinder adoption [46,47,49-51,55,67,68,77,79,85]. On the other hand, the users’ perception that the tool may ease the existing workload was also found to encourage adoption [52,53].

Workflow fit, which indicates how the tool would fit into and improve the day-to-day work of clinical staff, is another specific factor that was not defined as a distinct construct in most frameworks used, although it was mentioned in 12 studies [39,44,46,49,52,60,77,84,86]. Similarly, changes to clinical practices were specifically reported in nine studies, indicating that mHealth adoption sometimes required some process changes and adaptation to clinical practice, and this may hinder adoption if not addressed properly [15,41,45,49,53,71,77,87].

Compatibility (CFIR-DOI), adaptability (CFIR), practicability (APEASE), and job relevance (TAM2) were mentioned in eight studies and mostly related to the degree to which mHealth fits into, can be adapted, tailored, or refined to meet local needs [39,40,57,61,62,67,71,82]. Furthermore, four studies discussed a factor that was not specified as a distinct construct in most of the frameworks used, which is the impact of the perception of job security, job autonomy, perceived threat (dual-factor model), and empowerment. This latter factor shows that if clinicians perceive mHealth as a threat to their job autonomy or security or makes them feel that they are losing control of their work, they would resist its adoption [48,51,77,81].

We clustered the policy and regulations factors separately because they are external factors that go beyond the control of the organization. These are crucial in the health care setting, given their highly regulated nature but were hardly spelled out as specific constructs in most of the frameworks used, with the exception of CFIR. External policies (CFIR) and regulations were mentioned in eight studies and shed light on the importance of developing policies, clear guidelines, and legislative changes that would encourage adoption [15,35,39,53,62,76,78,85]. Reimbursement policies and funding were specifically mentioned in three studies [35,53,70], whereas two studies explained how the level of standardization in health care can facilitate or hinder adoption [58,78].

Patient-related factors were hardly defined as specific constructs in most of the frameworks used, despite the fact that various studies reported that they may impact the clinicians’ adoption of mHealth. Factors such as patient condition and whether they are capable of using the tool [47,51,53], their engagement, or lack of it [41,47,67,78], whether the tool facilitates patient empowerment [47,53], and whether it impacts patient safety [48] may influence a clinician’s decision on whether to adopt a specific mHealth tool. Furthermore, the tool’s availability and accessibility to all patients equally was mentioned in seven studies as a factor that may impact clinicians’ decision to adopt such a tool [35,40,47,52,55,70,82].

User engagement is another factor that was not defined as a distinct construct in the most frameworks used but specifically prevailed in five studies. The findings show that engaging the users in the design, development, and implementation of such tools can help encourage adoption [39,46,53,60,67].

## Discussion

### Framework-Based Versus Additional Factors

Expanded models allowed researchers in most of the included studies to examine potentially significant factors not specified in the original theories; nevertheless, some scholars have criticized such an approach of arbitrarily adding variables, as it leads to inconsistent use of theory [88].

Sometimes, the framework-based factors appear oversimplified or not precise enough. For example, if we say that usefulness is a key factor, we need to better understand the factors that influence the user’s perception of usefulness; without such specificity, it is difficult to take specific actions. This is perhaps why many of the included studies operationalized such variables by breaking them down into more specific elements, for example, by asking whether the tool was useful to the job or increased job efficiency. Such examples can be seen in studies by Gagnon et al [83] and Orruno et al [73], both of which assumed that compatibility with existing work practice would impact the perception of tool usefulness. Moreover, Liu and Cheng [81] anticipated that a user’s perception that a tool is a threat to job security would also impact their perception of usefulness. Similarly, Ray et al [45] proposed that there is, in fact, a whole cluster of contextual elements (organizational and individual factors) that influence perceived usefulness. The following sections discuss each of the frameworks used in more detail.

### Technology Acceptance Model and Unified Theory of Acceptance and Use of Technology Expansions

The TAM was used in 34% of the studied sample; Davis developed it in the late 1980s [89] based on the principles from Fishbein and Ajzen’s TRA [90]. The TAM assumes that perceived usefulness, and perceived ease of use are key predictors of the attitude toward using a new technology, which in turn determines the behavioral intention to use that can be translated into technology acceptance. Various scholars suggested extensions of the original TAM to overcome some of its limitations; notably, Holden and Karsh [91] suggested the addition of individual user factors, organizational readiness, or trust; Venkatesh and Davis [92] extended the model and referred to it as TAM2, which included factors that were considered to influence perceived usefulness, such as subjective norm, image, voluntariness of use, or job relevance. Venkatesh and Bala [93] extended the model further to TAM3 by adding computer anxiety or enjoyment.

There are many similarities between TAM and UTAUT, as the latter was based on the former. UTAUT was used in 12% of the sample; it was first published by Venkatesh et al [94] by analyzing and comparing TAM, TAM2, TRA, and DOI in an attempt to attain a unified TAM. The resulting model describes four key constructs:

1. Performance expectancy: the user’s belief that the tool will be useful for their job, which matches perceived usefulness in TAM.
2. Effort expectancy: the belief that the tool is user friendly and easy to use, which matches perceived ease of use in TAM.
3. Social influence: the degree to which users believe that important others think they should use the tool.
4. Facilitating conditions: the degree to which users think that an organizational and technical infrastructure exists to support the tool’s use.

Behavioral intention to use the technology is determined by performance expectancy, effort expectancy, and social influence. Actual usage is then determined by behavioral intention to use as well as the facilitating conditions. The model also adds moderating factors such as gender, age, experience, and voluntariness of use.

Most studies in our sample, where TAM or UTAUT were used, have added factors to extend the original frameworks to better adapt them to the context of health care. This demonstrates that despite their appeal, both frameworks frequently need some sort of extension to be applicable to complex health care settings. We briefly explain below the most significant expansion examples from our studied sample to emphasize the factors that researchers added to complement these 2 frameworks and their significance.

Orruno et al [73] and Gagnon et al [83] used very similar expansions of the TAM. They expanded the model with elements from the TIB and TRA to take into account the impact of the social environment and external variables as well as using Chau and Hu’s model [95] to further subdivide factors into individual, technological, and organizational contexts. They also added compatibility and technological context along with habit and facilitators as proposed by TIB to account for automatized behavior and organizational infrastructure, respectively. They also included subjective norms from the TRA to assess whether users believe that people who are important to them will approve of their adoption decision. Both papers showed that facilitators were the most significant factor in the modified model, thus emphasizing the importance of taking the organizational context into account.

Saigi-Rubio [74,75] used similar expansions in their two papers, one expanded the TAM with elements from the TPB and TRA and the other added elements from the DOI and technology readiness. In their first paper [75], they added three key factors: optimism (the degree to which clinicians consider that technology will enable them to obtain benefits or reduce effort), the propensity to innovate (an individual’s tendency to innovate in their daily work), and the level of information and communications technology (ICT) use (the degree to which an individual uses technology in their personal life). In their second paper [74], they also included personal technology use as a factor, calling it an *ICT user profile*, and added other factors such as security and confidentiality, improved quality, and reduced costs. They also considered the influence of patients, medical staff, and health care boards by assessing their impact on subjective norms. The papers showed that cost reduction, data security, and technology use in personal life were among the most important factors.

Asua [71] expanded the TAM with elements of the DOI and TIB; they justified this expansion with findings of some of their previous research, which showed that facilitating conditions were the most significant predictor of the clinicians’ intention to use telemonitoring. Their expansion confirmed that the facilitators in the organizational context are the most essential elements to consider for boosting mHealth adoption by clinicians. They categorized their constructs based on Chau and Hu’s model of telemedicine acceptance [95] that encompasses the individual, technological, and organizational contexts; whereas Ray et al [45] expanded the TAM using the organizational readiness for change model [96] and also shed some light on contextual factors. They added local elements such as meta-organizational, intraorganizational, and individual factors to their model. Their findings show that factors such as patient-specific education, clinical protocols for use, decreasing response times, and technology simplification may encourage adoption.

Liu and Cheng [81] expanded the TAM using the dual-factor model that considers both positive and negative factors by incorporating constructs that might hinder technological adoption, not only positive constructs such as TAM’s usefulness and ease of use. They were guided by Walter and Lopez [97], who suggested that the perceived threat to professional autonomy can influence the clinicians’ decision to adopt. They also added the factor of perceived mobility that was suggested by Huang et al [98], as they found that it can have a positive impact on perceived usefulness; this was also confirmed by Liu’s findings.

Rho et al [70] expanded the TAM based on previous research by adding three key factors: accessibility of medical records and of patients as clinical factors, self-efﬁcacy as an individual factor, and incentives as regulatory factors. Although Adenuga et al [66] expanded the UTAUT with the construct reinforcement factor to draw attention to the influence of direct incentives on adoption. In particular, financial incentives were reported as a significant factor in previous research, and their own research showed that reinforcement factors do indeed appear to have significant effects on the clinicians’ decision to adopt.

Dünnebeil et al [58] used a combination of UTAUT and TAM and expanded the model with additional factors that may impact perceived usefulness and perceived ease of use of electronic health (eHealth). They based their additions on factors that were identified as significant in most of the published research on TAM. They added factors that can impact usefulness, such as the intensity of IT utilization, the importance of data security, and the importance of documentation. They also identified factors that could influence ease of use, such as eHealth knowledge and the importance of standardization and process orientation. Similarly, Sezgin et al [82] used a combination of UTAUT and TAM and expanded them with elements from TPB and DOI. They added factors such as compatibility and personal innovativeness in the domain of IT from the DOI and computer self-efficacy and anxiety from TAM3. They also redefined some of the constructs to make them more suitable for mobile apps.

### Diffusion of Innovation Theory, Consolidated Framework for Implementation Research, and Sociotechnical Theory

In DOI theory, Rogers [99] goes beyond the technical aspects to argue that, ultimately, technologies that fit well into their context of use are more easily adopted than those that are not, it was the theoretical base for 16% of the studied sample and used together with TAM in another 6%. Rogers defines the five features of an innovation that will encourage its successful adoption; its relative advantage and the perception that it is better than the current processes, its compatibility with the adopters’ needs and experiences, its complexity and difficulty of use, its trialability, and the extent to which it can be experimented with on a limited basis, and the ease of the observability of its results.

The fact that the DOI takes the context into account makes it an attractive expansion choice to the TAM in as much as it complements and compensates for the TAM’s contextual gaps. In our sample, Putzer and Park [42,43] used elements from both frameworks in their two studies, guided by Kwon and Zmund, who suggested some modifications to the DOI with application research to make it more suitable for studying IT [100]. They added factors such as internal and external environment as well as moderating factors such as user demographics. They also removed trialability to reduce possible confusion with observability.

Other expansions of the DOI were also used. Han et al [76] expand the theory with Berwick’s model [101] that assumes that there are three groups of factors that influence adoption; first, the perception of whether the innovation is beneficial; second, the *types* of adopters themselves such as innovators, early adopters, early majority, late majority, and laggards; and third, contextual factors such as the social setting. Abd Ghani and Jaber [62] expand the theory with the technology-organization-environment model [102], as it provides a good understanding of adoption while balancing the flexibility of identifying specific factors emerging from individual contexts. This particular expansion also includes the social exchange theory [103], as it takes into account elements such as power and trust, shedding light on the importance of factors such as top management support. In addition, taken into account are technological, organizational, environmental, and individual characteristics.

The CFIR [104,105], used by 6% of the studies, goes even further to include implementation factors. It is a comprehensive framework with a list of 39 constructs, clustered in five key areas: features of the tool or intervention, characteristics of the users, qualities of the organization in which the intervention is implemented, the wider community and the social setting within which organizations function, and the implementation process itself. Given the extensive nature of this framework, it was used without expansion. Possemato et al [41] chose this framework because of its multidimensional nature that considers organizational and patient factors, and Varsi et al [67] used it because of its comprehensiveness, which enables it to capture the complexity of technology implementation in a health care setting and its ability to help identify barriers and facilitators that influence such implementations. However, despite its comprehensibility, our findings show that there were still some shortcomings, such as the lack of very specific factors such as interoperability, reimbursement, and data-related issues in the initial list of constructs.

Sociotechnical theory [106,107] focuses on how the social and technical aspects of a workplace fit together, and it was used in 4% of the studies. Ahmad et al [55] and Casey et al [49] chose it to help them investigate the factors that come into play when technological tools are put into practice. This framework views organizations as systems that include interconnected social and technical subsystems that interact together, thus necessitating the integration and coordination of social and technical elements to achieve joint optimization.

### Less Frequently Used Frameworks

There were also frameworks that only appeared in 1 publication. Nevertheless, many of these frameworks shed light on the importance of social and organizational aspects and employ theories that go beyond individual adoption decisions to also reflect the organizational context and the related implementation issues that may hinder adoption.

Various frameworks take into account the interactions that occur between the technical and social or organizational factors, highlighting the entanglement between those aspects. Bagot et al [15] employed the NPT [108,109], as it suggests a nonlinear understanding of adoption, taking into account the interdependent connections within an organizational context. The theory focuses on 4 key components: coherence (the process that people go through when attempting to understand a new set of practices), cognitive participation (the relational work that people do to build and sustain a new practice), collective action (the operational work that people do to enact a new practice), and reflexive monitoring (subsequent appraisal work undertaken to the effect of new practices). Their findings revealed that telemedicine necessitates both modifications in work practice and the development of new skills to enable successful implementation.

Similarly, Grünloh et al [77] wanted to not only understand clinicians’ views on technology but also its impact on their work environment; therefore, they used the TF model [110] focusing on the *assumptions, expectations, and knowledge* people use to comprehend technologies in their organizational context. In this framework, Orlikowski and Gash [110] recognized three key frames that can help explain users’ views: the nature of technology (users’ understanding of the technology and its functionality), the technology strategy (the motivation behind technology adoption and its worth to the organization), and technology in use (how the technology is being used on a daily basis and the implication of its use). The model helped the researchers identify work-related aspects such as the importance of processes, workload, and control (concerns that mHealth may lead to patients monitoring and controlling the physicians) when studying clinicians’ decision to adopt.

Some other frameworks had more emphasis on the role of the users and the inclusion of all relevant stakeholders. Bidmead and Marshall [47] used the Stakeholder Empowered Adoption Model [111] to ensure the inclusion of all relevant stakeholder groups in their study. The model categorizes stakeholders into four groups: professional users, patient users, organizational management, and technology providers. The study captured the views of all stakeholder groups to better understand barriers to adoption and to help each group recognize the perspectives of the other relevant stakeholders. Their findings showed that privacy concerns, risk aversion, and data integration issues are the key barriers hindering adoption. Kuo et al [80] employed the TPB [112] using three key factors: attitude (users’ feelings about using telemedicine), subjective norms (whether the user should or should not use telemedicine), and perceived behavioral control (the availability of the skills, opportunities, and resources required for using telemedicine). Their results show that the three factors have an impact on technological adoption.

Sharma et al [51] used Gidden’s structuration theory [113-115] to help them tackle issues such as trust and ontological security, which are not really addressed in most other frameworks. They were guided in their thinking by Kouroubali’s argument that a better understanding of such social aspects can help resolve potential conflict and contradiction, and enable successful implementation [116]. Their findings emphasized some key barriers such as insufficient training, support, and lack of information, highlighting the importance of gaining clinicians’ trust and promoting a sense of security to encourage telehealth adoption.

Bramley et al [48] took a different approach by looking into the potential for change in the organizational culture that they were examining. They employed the theory of change [117] and investigated the factors that would help the key stakeholders be less resistant to change. They focused on three levels of stakeholders: the macrolevel, which focuses on how commissioning authorities work with technology providers and other organizations; the mesolevel, comprising providers working with clinicians and health care providers; and the microlevel, in which the cocreation and delivery of care packages occur. Their findings showed that elements such as staff buy-in, culture, privacy concerns, safety issues, leadership engagement, data analysis, and local setting are crucial for successful implementation. Similarly, Shaw et al [46] addressed the topic from an organizational change perspective employing the Weiner organizational theory of implementation effectiveness [96,118]. The model can help identify the factors impacting an organization’s readiness for change, which refers to its members’ collective agreement and the ability to implement a specific change. Such readiness depends on two key concepts: change valence, which refers to the organization’s perception of factors such as the fit, advantage, and need for this change and informational assessment, which refers to elements such as information about the workload needs; and the availability of resources to implement the change. The study identified organizational barriers related to additional workload, lack of resources, and workflow integration.

Furthermore, some theories had more focus on the design process itself. Egerton et al [52] used the APEASE framework [119], which is usually used to evaluate the design of new interventions and services. Their study identified some adoption barriers related to lack of information and support, disconnect with other programs, sense of loss of control, and a lack of familiarity with the new tool, resulting in resistance to change. Lapão [85], on the other hand, employed the DSRM [120,121] to help them study the link between research and practice by designing, implementing, and evaluating a tool that tackles a specific need. The methodology starts with problem identification and then moves through the definition of the objectives that result in the design and development of the tool, followed by testing and evaluation. Their findings show that the key barriers for implementation are the lack of time, skills, and clear role definition.

Quanbeck et al [44] focused on the implementation process itself and used the RE-AIM framework [122] to achieve that. They identified factors such as system integration, cost, and data management as key barriers for implementation. Furthermore, Okazaki et al [63] expanded the DeLone and McLean information system success model [123] to help them assess the success of a new technology in a specific organizational context. They used the original constructs of system quality, information quality, service quality, net benefits, and user satisfaction and added some additional factors such as privacy and security risks, ubiquitous control (time and place flexibility), and subjective norms from TPB. Their results showed that perceived value and net benefits are the most important factors impacting adoption.

### Conclusions and Recommendations

**Figure 5:**
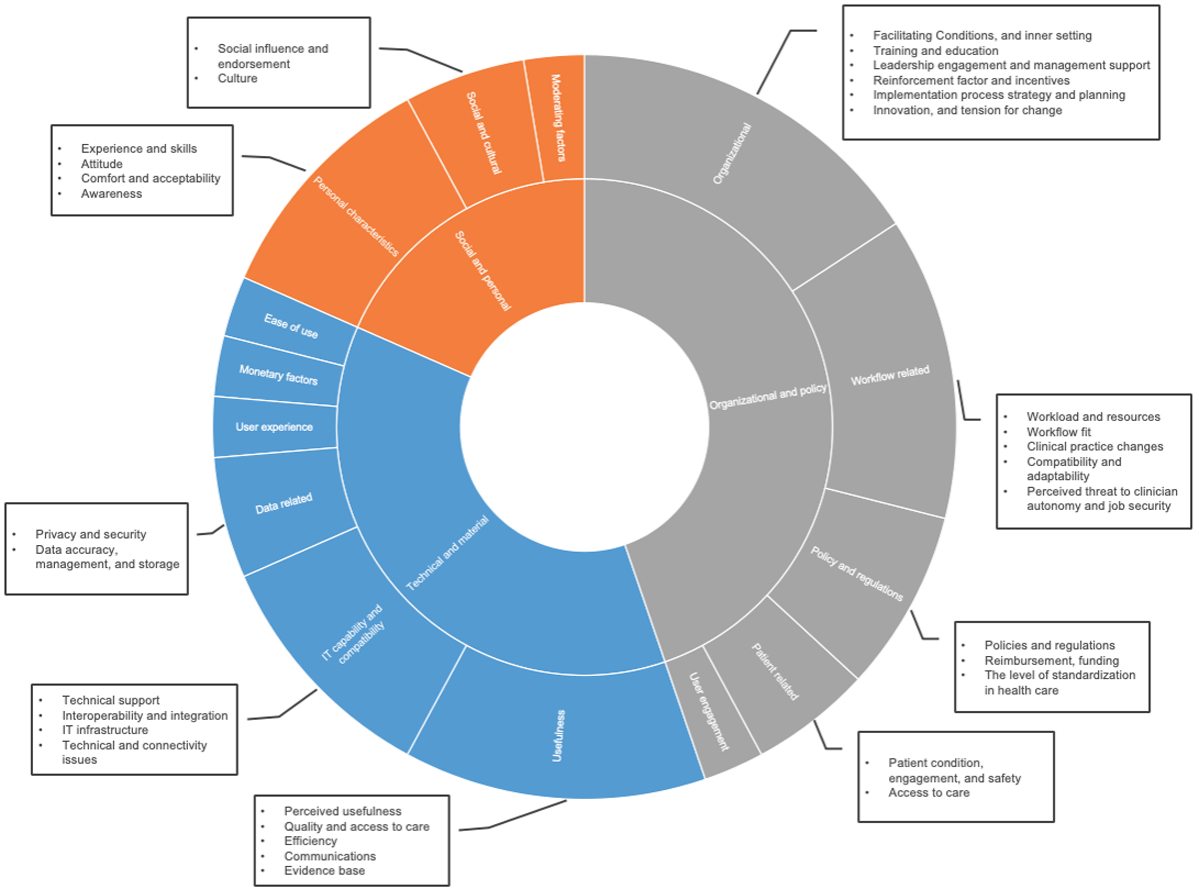
Consolidated framework of the factors impacting clinicians' adaptation of mobile health. IT: information technology.

Health care technologies are generally more complex than tools that address one specific user need, as they usually support patients with comorbidities that are typically treated by multidisciplinary teams, potentially working across more than one health care organization. The specific characteristics of how the health care sector operates (its highly regulated nature, ever-present budget deficits, and the interdependence between health care organizations) necessitate some crucial extensions to the existing generic frameworks for studying adoption. This paper sheds light on these specificities and makes recommendations regarding the important factors that should not be overlooked when working on mHealth adoption in health care. These include stringent data privacy, workload, workflow, communication, management support, policies, and complex external rules and regulations, as presented in our consolidated framework of emerging framework-based and additional factors in Figure 5.

Most factors can be related to one framework or another, but no framework covers all factor categories without being extended. Although most models include relevant constructs, many of them do not break these down into their individual components to enable researchers to examine the specific reasons behind particular implementation or adoption issues and so tend not to help in the identification of specific solutions.

Some of the commonly used frameworks, such as TAM, present an oversimplified group of factors that need to be looked at in much more detail, and with greater specificity, if we are to develop and implement successful mHealth tools. Although it is sometimes claimed that it is simplicity that makes such frameworks useful [124], even in the health care context [91,125,126], our conclusions are aligned with the findings of researchers’ critical frameworks such as TAM and UTAUT for overemphasizing individual user beliefs and perceptions [127]. Most of these frameworks were not developed within a health care setting and thus overlook its organizational and regulatory complexity [91,128,129]. Socio-organizational and cultural factors are not well covered by frameworks such as TAM and UTAUT [128,129]. We also note that many of the broadly used frameworks focus on tools that can be voluntarily used by individual adopters. In contrast, most health care settings involve an organizational-level decision to roll out technologies that are made available to all staff [128,130].

We propose a shift toward extended frameworks that take into account the complexity of health care organizations, their highly regulated nature, their interdependence, and the active role of the user in impacting how technology is being used in the work environment. Other researchers have also cited the technology-centered view of many of the broadly used models that mostly focus on the tool itself [128,129,131] and suggested a move to multidimensional theories that go beyond usability and consider the surrounding contexts and implementation issues [127,128,131-134]. Health care technology cannot be successfully adopted in isolation from the broader organizational context in which it is being used; therefore, we need to adopt theoretical frameworks that consider implementation challenges in light of the complexity of the sociotechnical structure and the interplay between the technical, social, and organizational aspects. This can be achieved by using our suggested consolidated framework that highlights the existing gaps that are not specifically covered in the most frequently used frameworks and focuses on complementing them using a sociotechnical approach that enables researchers to take all the contextual factors, and, crucially, the interplay between them, into account when studying adoption.

### Limitations and Future Research

Although this study contributes to the understanding of the most used frameworks in understanding clinicians’ adoption of mHealth, certain limitations must be acknowledged. This review may not have included related studies that were not indexed in the searched databases, written in a language other than English, and gray literature searches that could have also permitted the identification of further relevant insights. However, this study focused on peer-reviewed scientific papers.

Moreover, this analysis only considered published studies, and no further contacts were made with the authors of the papers to obtain extra information or to validate our thematic analysis. Consequently, it is possible that other frameworks might have been missed. Future reviews could include studies in other languages and using other frameworks.

## Appendix

Multimedia Appendix 1 Phases of thematic analysis.

Multimedia Appendix 2 Summary of the included studies.

Multimedia Appendix 3 Characteristics of the included studies.

Multimedia Appendix 4 Overview of the most used frameworks.

Multimedia Appendix 5 Definitions of the most prevalent framework-based themes.

## Abbreviations

APEASE: affordability, practicability, effectiveness, acceptability, safety/side effects, and equity
CFIR: consolidated framework for implementation research
DOI: diffusion of innovation theory
DSRM: design science research methodology
eHealth: electronic health
IT: information technology
mHealth: mobile health
NPT: normalization process theory
STS: sociotechnical system
TAM: Technology Acceptance Model
TF: technological frames
TIB: theory of interpersonal behavior
TRA: theory of reasoned action
UTAUT: unified theory of acceptance and use of technology
