# Supplementary figures and images for: Understanding Clinicians’ Adoption of Mobile Health Tools: A Qualitative Review of the Most Used Frameworks
Source: JMIR Mhealth Uhealth. 2020 Jul 6;8(7):e18072. doi: 10.2196/18072 (PMC7381026; doi:10.2196/18072)

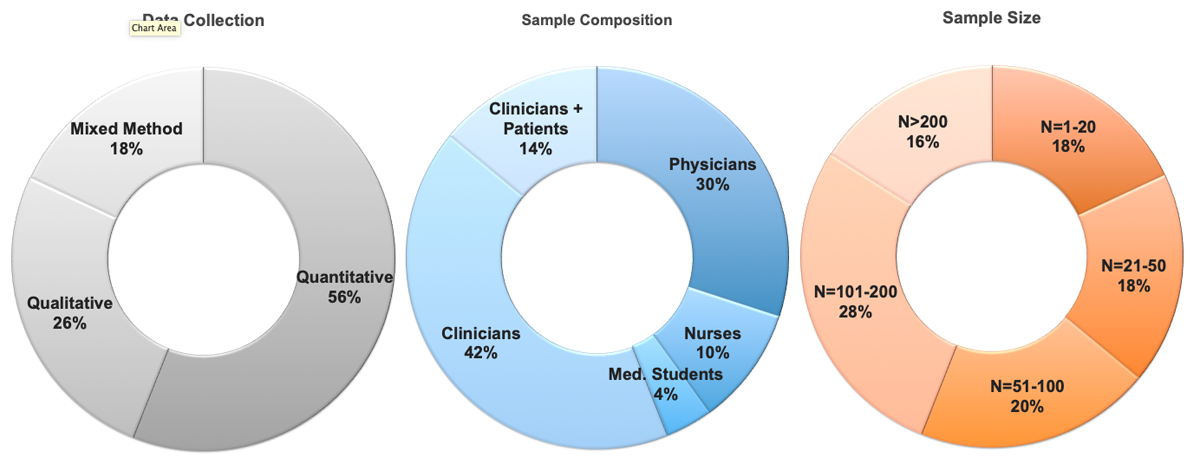

Supplement: Multimedia Appendix 3 [file mhealth_v8i7e18072_app3.png]

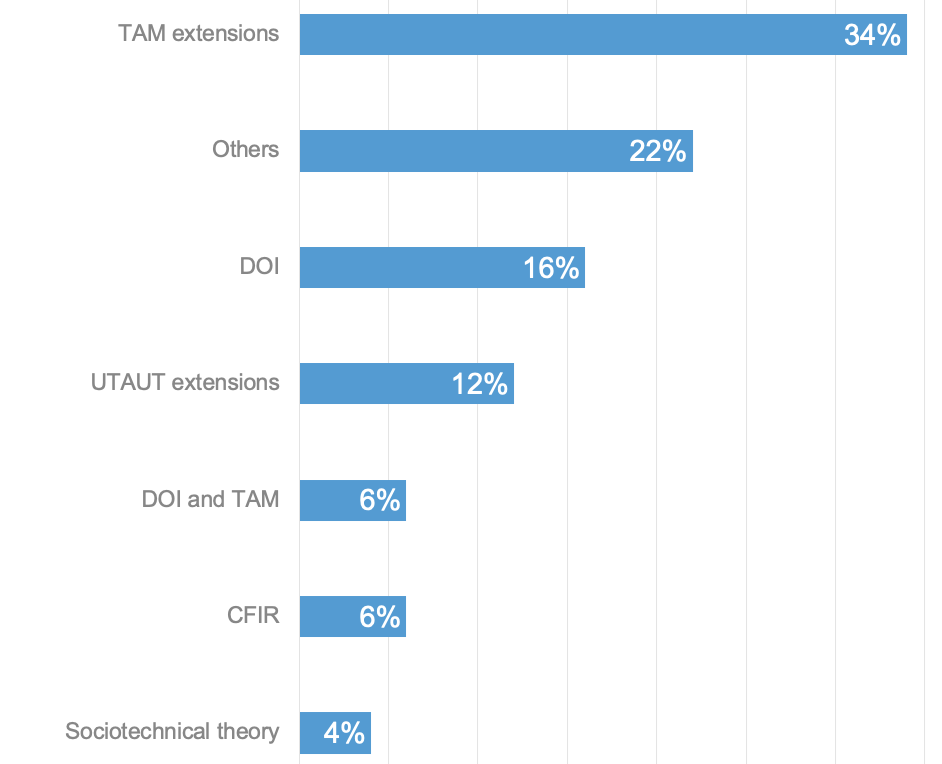

Supplement: Multimedia Appendix 4 [file mhealth_v8i7e18072_app4.png]
