# Supplementary material for: Understanding Clinicians’ Adoption of Mobile Health Tools: A Qualitative Review of the Most Used Frameworks
Source: JMIR Mhealth Uhealth. 2020 Jul 6;8(7):e18072. doi: 10.2196/18072 (PMC7381026; doi:10.2196/18072)
Supplement: Multimedia Appendix 5 [file mhealth_v8i7e18072_app5.pdf]

Multimedia Appendix 4. Definitions of the most prevalent framework-based themes.

Subtheme and factor (framework)	Definition
**Technical and material factors**	
**Usefulness**	
Perceived usefulness (TAM[a])	The usefulness the individual sees in using the technology
Performance expectancy (UTAUT[b])	The perception a user has that the technology has inherent benefits
Relative advantage (CFIR[c]-DOI[d])	Stakeholders' perception of the advantage of implementing the intervention versus an alternative solution. The degree to which the innovation is perceived as better than the existing process
Output quality (TAM2[e])	The perceived system's output
Effectiveness (APEASE[f])	The degree to which the tool is successful in producing a desired result; success
Evidence (CFIR)	Stakeholders' perceptions of the quality and validity of evidence supporting the belief that the intervention will have desired outcomes
**Ease of use**	
Perceived ease of use (TAM)	The perceived ease of using the technology
Effort expectancy (UTAUT)	The perception a user has that the technology is easy to use
Complexity (CFIR-DOI)	Perceived difficulty of the intervention, reflected by duration, scope, radicalness, disruptiveness, centrality, and intricacy and number of steps required to implement. The degree to which the innovation is perceived as being difficult to understand and use
**Monetary factors**	
Cost (CFIR)	Costs of the intervention and costs associated with implementing the intervention including investment, supply, and opportunity costs
Affordability (APEASE)	Cost and funding
**User experience**	
Design quality (CFIR)	Perceived excellence in how the intervention is bundled, presented, and assembled
Source (CFIR)	Perception of key stakeholders about whether the intervention is externally or internally developed
**Social and personal factors**	
**Personal characteristics**	
Self-efficacy (CFIR)	Individual belief in their own capabilities to execute courses of action to achieve implementation goals
Attitude (TAM-TPB[g])	The perception of the positive or negative consequences related to adopting the technology. Positive or negative feelings about using telemedicine
Habit (TIB[h])	Behavior that has become automatized
**Social and cultural factors**	
Social influence (UTAUT)	The influence of others on a prospective technology adopter
Observability (DOI)	The easier it is for individuals to see results of the innovation, the more likely they will be to adopt it

		Subjective norm (TPB-TRA[i])	The extent to which an individual believes that people who are important to him or her will approve his or her adopting of a particular behavior
		Culture (CFIR)	Norms, values, and basic assumptions of a given organization
**Organizational and policy factors**			
	**Organizational factors**		
		Facilitating condition (TIB-UTAUT)	The perception a user has that organizational support and technical facilitating conditions are in place to aid technology use
		Perceived behavioral control (TPB)	Perceptions of the availability of skills, opportunities, and resources required for using telemedicine
		Inner setting (CFIR)	Qualities of the organization in which the intervention is implemented
		Leadership engagement (CFIR)	Commitment, involvement, and accountability of leaders and managers with the implementation
		Incentives (CFIR)	Extrinsic incentives such as goal-sharing awards, performance reviews, promotions, and raises in salary and less tangible incentives such as increased stature or respect
		Process (CFIR)	The implementation process, strategy, and planning
		Tension for change (CFIR)	The degree to which stakeholders perceive the current situation as intolerable or needing change
	**Workflow-related factors**		
		Available resources (CFIR)	The level of resources dedicated for implementation and on-going operations, including money, training, education, physical space, and time
		Compatibility (CFIR-DOI)	The degree of tangible fit between meaning and values attached to the intervention by involved individuals, how those align with individuals' own norms, values, and perceived risks and needs, and how the intervention fits with existing workflows and systems
		Adaptability (CFIR)	The degree to which an intervention can be adapted, tailored, refined, or reinvented to meet local needs
		Practicability (APEASE)	The quality of being practicable; viability
		Job relevance (TAM2)	The importance of the technology for the job
		Perceived threat (dual-factor model)	Walter and Lopez [86] introduced this new negative factor of "perceived threat to professional autonomy" based on the dual-factor model
	**Policy and regulations**		
		External policies (CFIR)	A broad construct that includes external strategies to spread interventions, including policy and regulations (governmental or other central entity), external mandates, recommendations and guidelines, pay-for-performance, collaboratives, and public or benchmark reporting

[a]TAM: Technology Acceptance Model.

[b]UTAUT: unified theory of acceptance and use of technology.

[c]CFIR: consolidated framework for implementation research.

[d]DOI: diffusion of innovation theory.

[e]TAM2: an expanded version of the original TAM.

[f]APEASE: affordability, practicability, effectiveness, acceptability, safety/side effects, and equity.

[g]TPB: theory of planned behavior.

[h]TIB: theory of interpersonal behavior.

[i]TRA: theory of reasoned action.
